# Gene regulatory network underlying the immortalization of epithelial cells

**DOI:** 10.1186/s12918-017-0393-5

**Published:** 2017-02-16

**Authors:** Luis Fernando Méndez-López, Jose Davila-Velderrain, Elisa Domínguez-Hüttinger, Christian Enríquez-Olguín, Juan Carlos Martínez-García, Elena R. Alvarez-Buylla

**Affiliations:** 1 Instituto de Ecología, UNAM, Cd. Universitaria, México, 04510 D.F México; 2 Centro de Ciencias de la Complejidad, UNAM, Cd. Universitaria, México, 04510 D.F México; 3 Departamento de Control Automático, Cinvestav-IPN, A. P. 14-740, México, 07300 D.F México; 4 Centro de Investigación y Desarrollo en Ciencias de la Salud (CIDICS), Universidad Autonoma de Nuevo Leon, A. P. 14-740, México, 07300 D.F México

**Keywords:** Carcinomas, Gene regulatory networks, Epigenetic landscape, Boolean models, Phenotypic attractors

## Abstract

**Background:**

Tumorigenic transformation of human epithelial cells in vitro has been described experimentally as the potential result of *spontaneous immortalization*. This process is characterized by a series of cell–state transitions, in which normal epithelial cells acquire first a senescent state which is later surpassed to attain a mesenchymal stem–like phenotype with a potentially tumorigenic behavior. In this paper we aim to provide a system–level mechanistic explanation to the emergence of these cell types, and to the time–ordered transition patterns that are common to neoplasias of epithelial origin. To this end, we first integrate published functional and well–curated molecular data of the components and interactions that have been found to be involved in such cell states and transitions into a network of 41 molecular components. We then reduce this initial network by removing simple mediators (i.e., linear pathways), and formalize the resulting regulatory core into logical rules that govern the dynamics of each of the network components as a function of the states of its regulators.

**Results:**

Computational dynamic analysis shows that our proposed Gene Regulatory Network model recovers exactly three attractors, each of them defined by a specific gene expression profile that corresponds to the epithelial, senescent, and mesenchymal stem–like cellular phenotypes, respectively. We show that although a mesenchymal stem–like state can be attained even under unperturbed physiological conditions, the likelihood of converging to this state is increased when pro–inflammatory conditions are simulated, providing a systems–level mechanistic explanation for the carcinogenic role of chronic inflammatory conditions observed in the clinic. We also found that the regulatory core yields an epigenetic landscape that restricts temporal patterns of progression between the steady states, such that recovered patterns resemble the time–ordered transitions observed during the spontaneous immortalization of epithelial cells, both in vivo and in vitro.

**Conclusion:**

Our study strongly suggests that the in vitro tumorigenic transformation of epithelial cells, which strongly correlates with the patterns observed during the pathological progression of epithelial carcinogenesis in vivo, emerges from underlying regulatory networks involved in epithelial trans–differentiation during development.

**Electronic supplementary material:**

The online version of this article (doi:10.1186/s12918-017-0393-5) contains supplementary material, which is available to authorized users.

## Background

Nearly 84% of cancers diagnosed in human adults are carcinomas (i.e., cancer of epithelial origin). Although epithelial carcinogenesis has been strongly associated with a chronic inflammatory process and aging [1], the precise role of these two processes to the origin and progression of carcinomas remains elusive. The current general assumption is that aging and inflammation increase the chance of accumulating somatic mutations, and that this genetic instability constitutes the cause of carcinoma. But this view does not explain several well–described experimental and clinical observations. For instance, cancer behavior can be acquired in the absence of mutations through trans–or de–differentiation and is characterized for recapitulating embryonic processes. Cancer cells can be “normalized” by several experimental means and commonly show morphological and transcriptional convergence despite their diverse origin and mutations [2–4]. In addition, different carcinomas share similar time–ordered patterns of progression, as well as clear associations with lifestyle factors in many cases [5]. These empirical observations suggest that, in analogy to normal development, epithelial carcinogenesis is a consequence of conserved or generic system–level mechanisms that restrict the malignant phenotypes that can be attained, as well as the temporal patterns of progression that describe the transitions between them. In accordance with this latter view, it has been proposed that cancer can be considered a developmental disease [6].

In agreement with this developmental view of cancer, numerous experimental findings in molecular and cell biology of cancer research have revealed that it is possible to recover cells with cancer–like phenotypes through the induction of de–differentiation events. This has been shown particularly in carcinomas [3, 7], since inflammatory cytokines induce a de–differentiation event of epithelial cells denominated Epithelial–Mesenchymal Transition (EMT) in which cells acquire a mesenchymal stem–like phenotype with a tumorigenic potential able to develop cancer in mice [3].

In systems biology it is common to study cell differentiation processes that underlie development and pathogenesis from the point of view of dynamical systems theory. In this framework, the information encoded by the genome, in addition to epigenetic mechanisms, can be mapped to a gene regulatory network (GRN), that shows multiple stable steady states, each of which corresponds to a particular phenotypic cellular state. Further, the GRN also underlies the epigenetic landscape (EL), that restricts the time–ordered patterns of transition between the phenotypes [8–13]. Thus, the same genome and GRN can robustly generate multiple discrete cellular phenotypes through developmental dynamics [11, 14, 15]. These stable phenotypic states are called *attractors* and correspond to configurations of gene or protein activation states that underlie the cellular fates or phenotypes. Therefore, dynamic developmental processes – particularly, cellular differentiation and morphogenetic patterning – can be formalized in temporal terms as transitions between attractors (i.e., cell states). Here, we adopt such approach to study how tumorigenic transformation due to spontaneous immortalization via EMT emerges from the regulatory interactions between different molecular players that are known to contribute to the tumorigenic transformation of epithelial cells. We hypothesize that: (1) a generic series of cell state transitions describing the phenotypic transformation of epithelial cells first to senescent cells and finally to mesenchymal stem–like cells, that is widely observed in epithelial cell cultures during spontaneous immortalization, naturally result from the self–organized behavior emerging from an underlying intracellular GRN; and (2) that pro–inflammatory tissue–level conditions, which are associated with a bad prognosis, increase the likelihood of this tumorigenic process, promoting the emergence and progression of epithelial cancer.

To test our hypothesis, we propose here a cellular–level GRN that, for the first time, integrates those molecular components and their interactions that have been experimentally shown to play an important role during the emergence and progression of carcinomas. It includes the key molecular regulators that: (1) characterize the cellular phenotypes of epithelial, mesenchymal stem–like, and senescent cells; (2) are involved in the induction of the cellular processes of replicative senescence, cellular inflammation, and epithelial–mesenchymal transition (EMT); and (3) are involved in the phenotypic changes undergone by cells emerging from these processes (i.e., mesenchymal stem–like cells). We then obtained a reduced regulatory core for further dynamical analyses by removing linear cascades while maintaining the feedback loops. We show that the proposed regulatory core module displays an orchestrating robust behavior akin to that seen in other developmental regulatory modules previously characterized with similar modeling approaches (see, for example [8, 9, 16, 17]). Specifically, by proposing logical functions grounded on the available experimental data for this regulatory core module, and by analyzing its behavior following conventional Boolean GRN dynamical approaches, we show that the uncovered GRN converges to exactly the three attractors corresponding to the expected gene expression configurations characterizing the epithelial, senescent, and mesenchymal stem-like phenotypes. Additionally, using a stochastic version of the model to explore the GRN EL (following the methodology proposed in [13]), we found that the proposed GRN also explains the commonly observed temporal sequence by which epithelial cells acquire the potentially tumorigenic mesenchymal stem–like phenotype.

Our results suggest that the proposed core GRN incorporates a set of necessary and sufficient components and interactions to explain the emergence of gene configurations characteristic of epithelial, senescent and mesenchymal cells, as well as the time–ordered sequence of cellular transformations observed in the spontaneous immortalization process that, in turn, underlies the tumorigenic transformation of epithelial cells.

## Results

### Gene regulatory network underlying spontaneous immortalization

**Fig. 1:**
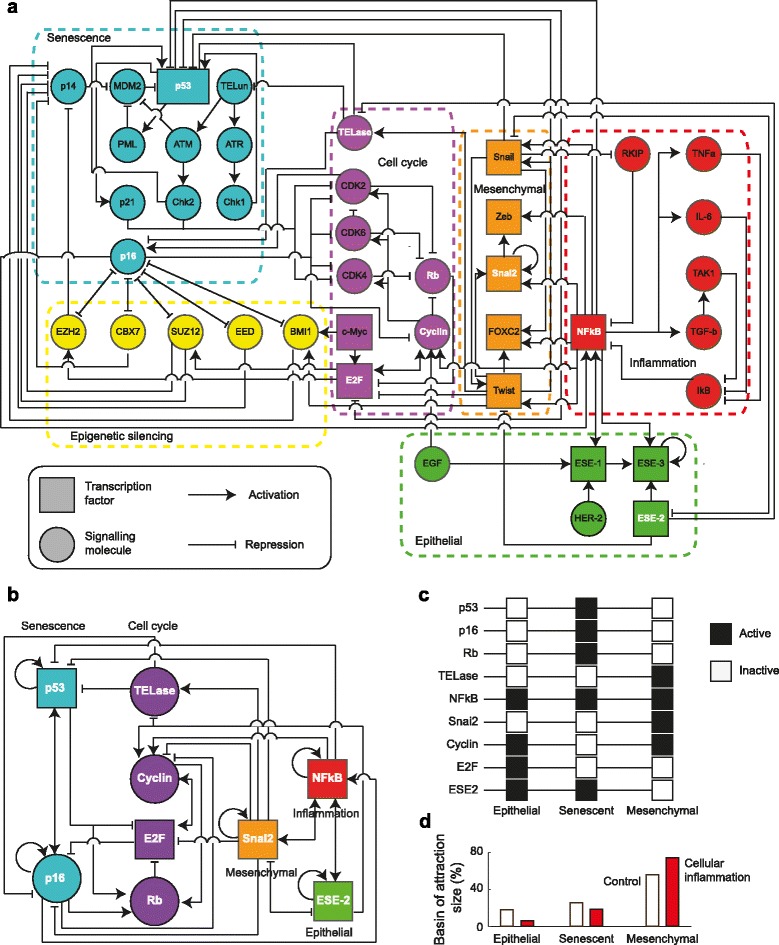
Gene Regulatory Network underlying spontaneous immortalization. **a** Gene regulatory network for epithelial carcinogenesis. **b** A Core Regulatory Network Module Underlying Spontaneous Immortalization and Epithelial–Mesenchymal Transition. **c** Predicted gene expression profiles characterizing the epithelial, senescent and mesenchymal stem–like cells. **d** Cellular inflammation increases the size of the basin of attraction of the mesenchymal stem–like phenotype

Following a bottom–up approach, we performed an extensive literature search to gather the most relevant experimental functional molecular data describing the cellular–level processes involved in epithelial carcinogenesis, namely: replicative cellular senescence, inflammation, and EMT (see Additional file 1: Table S4). We found a set of 41 molecular players (12 TFs and 29 signaling molecules) which are involved in epithelial or mesenchymal cell differentiation, cellular inflammation, senescence, DNA damage, cell cycle, or in epigenetic silencing; as well as 97 regulatory interactions between them. For the first time, we integrated this previously scattered experimental information into the GRN represented in Fig. 1 a. To further support that the set of regulatory interactions that we manually curated based on published data are indeed representative of the cellular–level processes underlying epithelial carcinogenesis, we performed a network–based Gene Set Enrichment Analysis (GSEA) of the GRN, using both the KEGG and the GO Biological Process databases as reference. We found that among the 12 pathways or processes reported as significant when taking the KEGG database as a reference, 10 (≈ 83%) correspond to the cancer types bladder cancer, chronic myeloid leukemia, non-small cell lung cancer, glioma, melanoma, pancreatic cancer, prostate cancer, small cell lung cancer, thyroid cancer, from which 6 (66.6%) correspond to carcinomas. When taking the GO Biological Process database as reference, we found that the molecules considered in our regulatory network are significantly enriched for several of the biological processes known to play important roles during spontaneous immortalization of epithelial cells, namely replicative senescence, cellular senescence, cell aging, positive regulation of ephitelial to mesenchymal transition determination of adult life span, among others (Table 1). Additionally to these GSEA, we performed a Network-based topological gene set enrichment analysis (described in Methods) and found that, in addition to the enrichment of the pathways and processes described above, the molecules in the proposed network show also a topological signature that strongly resembles the structure of the cancer pathways included in the KEGG database (see Additional file 2: Figure S1). The complete enrichment results are included in Additional file 3: Supplementary Tables. These results provide further support for the relevance of the proposed molecular players, manually assembled from an exhaustive literature search, and for the novel regulatory module proposed here.

**Table 1:** Most significant pathways and processes in the GRN (Fig. 1 a) shown by a network–based gene set enrichment analysis

KEGG – Pathway or Process (*carcinomas marked in italics*)	Functional association (XD–score)	q-value	Overlap/Size
*Bladder cancer*	1.06805	0	11/38
Chronic myeloid leukemia	0.66539	0	13/69
p53 signaling pathway	0.68435	0	12/62
*Pancreatic cancer*	0.53872	0	11/70
Glioma	0.57682	0	10/60
*Non–small cell lung cancer*	0.61604	0	9/51
*Melanoma*	0.55531	0	10/62
*Small cell lung cancer*	0.39796	0	10/82
*Prostate cancer*	0.43396	0	11/84
Cell cycle	0.44349	0	16/120
Cytosolic DNA–sensing pathway	0.48155	0.00001	6/40
Thyroid cancer	0.39015	0.00564	3/25
*Endometrial cancer*	0.31015	0.00018	5/50
GO Biological Process			
*Replicative senescence*	3.138	0	8/10
Cellular senescence	0.738	0.01815	2/10
Cell aging	0.438	0.00461	3/24
*Positive regulation of epithelial to mesenchymal transition*	0.438	0.03501	2/16
Determination of adult lifespan	0.33328	0.40382	1/10

In conclusion, based on these analyses and the current state of knowledge according to annotated databases, the set of molecules manually included in the proposed large network seem to be representative of the cellular phenotypes and processes considered as prior biological knowledge in our model. In addition, the molecular components included in the proposed network are tightly associated with reference pathways of epithelial cancers. Additionally, robustness analysis performed on the network showed that the recovered attractors are also robust to permanent alterations of the regulatory logic (see Additional file 4: Text).

To study the dynamic and steady–state properties of the network underlying epithelial carcinogenesis, we reduced the initial network (Fig. 1 a) into a more mathematically and computationally tractable regulatory core network by collapsing the linear pathways while retaining the feedback loops. This reduced network, represented in Fig. 1 b, retains only the main players and regulatory interactions that underlie epithelial and mesenchymal differentiation, cell cycle progression, senescence, and cellular inflammation.

*Epithelial differentiation* is represented by the tumor suppressor Epithelium-Specific TF ESE–2, which acts as a master regulator of this process by triggering the expression of epithelium-specific genes while repressing mesenchymal markers, such as Snail [18]. ESE–2 also promotes its own expression and the expression of the other ESE TFs [19], and it is hence considered as the main representative of the TF family [18]. The differentiation of epithelial cells into a *mesenchymal stem–like phenotype* is orchestrated by the TF Snai2, which further induces its own expression via the activation of Twist-Zeb-FOXC2 [20]. The progression of the *cell cycle* is controlled by Rb, E2F, Cyclin, and Telomerase (here TELase). While both E2F [21] and cyclins [22] are required for cell cycle progression, the tumor suppressor Rb acts as an inhibitor of this process by forming an inactive heteromer with E2F [23]. The enzyme TELase is responsible for the de novo synthesis of telomeres, a process that allows cells to surpass the cell cycle checkpoints and become immortalized [24]. Indeed, high levels of TELase are typical of tumor initiating cells that become resistant to therapy [3, 25]. The *senescence* of epithelial cells is mainly controlled by the two tumor supressors p53 and p16 [26]. They both induce replicative senescence by reducing the activity of cyclins [27] and by inducing an Rb-mediated inhibition of E2F [28]. *Cellular inflammation* is characterized by the increased activity of the TF NF– *κ*B. Many (micro)environmental stimuli, including pathogens, cytokines, interleukins, and antigens; trigger the expression of NF– *κ*B, resulting in an immune response characterized by increased levels of cytokines and enzymes such as phospholipase A2, cyclooxygenase, and lipoxygenase [29].

The above described molecular players (ESE–2, Snai2, Rb, E2F, cyclin, TELase, p53, p16, and NF– *κ*B) are tightly interconnected by the following regulatory interactions: *NF–* *κ* *B* positively controls its own expression via the induction of pro-inflammatory cytokines [30, 31]. It also promotes the epithelial and mesenchymal stem–like phenotype by positive interactions with ESE–2 [32] and Snai2 [33], respectively; and it induces the cell cycle process by increasing cyclin [34] and inhibiting p53 expression [35]. *ESE–2* forms an auto–activation feedback loop, inhibits Snai2 [18], induces cyclin, inhibits TELase [18], and activates of NF– *κ*B [36]. *Snai2* induces its own expression [37], increases the activity of TELase [38], and decrease the expression of the epithelial–specific TF ESE–2 [18]. It also represses the cell cycle process by reducing the transcription of cyclin [39] and E2F [40], represses cellular senescence by decreasing the expression of p16 [41] and p53 [42], and induces cellular inflammation by increasing the expression of NF– *κ*B [43]. *p16* promotes its own activity forming a positive feedback loop [44], stabilizes p53 via the inactivation of its inhibitor MDM2 [45], and inhibits the cell cycle progression by the induction of Rb [46] and the inhibition of cyclin [23, 28]. These molecular processes are known to induce the senescent phenotype. p16 also contributes to the cellular inflammation observed in senescent cells by inducing the activation of NF– *κ*B [47]. *p53* inhibits E2F [48]. *Rb* inhibits E2F [23]. *E2F* induces cyclin [49] and inhibits the senescence marker p16 [50]. *Cyclin* inhibits Rb activity [51] and stimulates E2F transcription [52]. Finally, *TELase* inhibits the senescence markers p16 [53] and p53 [54].

To analyze its long term and dynamic properties, we then formalized the above described regulatory interactions (Fig. 1 b), by translating the nodes and their corresponding logical interaction rules into a mechanistic Boolean dynamical GRN model [55]. The corresponding logical rules and truth tables can be found in the Additional file 5: Logic rules and truth tables.

### The epithelial, senescent, and mesenchymal stem–like phenotypes are attractors of the GRN

It has been experimentally shown that during the process of spontaneous immortalization epithelial cells acquire first a senescent and finally a mesenchymal stem–like cell phenotype [56], each of which can be described in terms of their gene expression profiles. Here, we performed long-term (technical term) simulations of our Boolean model of the GRN with the aim to recover these three characteristic expression patterns. We found that indeed our GRN (Fig. 1 b) converges to three attractors, each corresponding, respectively, to the epithelial, senescent, and mesenchymal stem–like phenotypic markers (Fig. 1 c).

*Epithelial cells* are characterized by the high expression of the TF ESE–2, which acts as a master regulator. Being part of a constantly renewing tissue, epithelial cells show an increased expression of the cell cycle inducers Cyclin and E2F and a decreased expression of the cell cycle inhibitor Rb [57] and of the senescence markers p53 and p16 [58]. Epithelial cells do not express TELase, since the activity of this enzyme is inhibited in response to induction of differentiation events [59]. Being a constitutively expressed gene, epithelial cells show also high levels of the TF NF– *κ*B [60]. In accordance to this empirical activity profile, approximated as a Boolean vector, the first attractor of our GRN shows an absence of activity for p53, p16, Rb, TELase and Snai2; and presence of NF– *κ*B, Cyclin, E2F and ESE–2 (Fig. 1 c, left).

*Senescent* epithelial cells conserve the high levels of the epithelial markers ESE–2 [61] and the low expression of the mesenchymal marker Snai2 [18]; but, in contrast to normal epithelial cells, these cells show an increased expression of the two tumor suppressor proteins p16 and p53 [62], both of which contribute to the cell cycle arrest by inhibiting the cell cycle inducers Cyclins and E2F [27, 28, 63] and by inducing the cell cycle suppressor Rb [64]. As in homeostatically cycling epithelial cells, TELase is inactive in this cell type [59]. Also in this case, NF– *κ*B expression is constitutively active. These features are recovered in our second attractor, which shows activity of NF– *κ*B, p53, p16, Rb and ESE2, and absence of TELase, Snai2, cyclin, and E2F (Fig. 1 c, center). Also in this case, our in silico analysis predicts activity of NF– *κ*B in epithelial senescent cells.

*Mesenchymal stem–like* cells express the mesenchymal marker Snai2 [18], which acts as one of the key players during the EMT by orchestrating the repression of epithelium–specific genes [65] such as ESE–2 [18] and by inducing the expression of mesenchymal markers [63]. In contrast to the senescent cells, these mesenchymal stem-like cells have a strong proliferative potential, shown by a decreased expression of p53, p16, Rb; and by a high expression of cyclin [66]. Cell cycle progression is further promoted in these potentially tumor initiating cells by the high levels of TELase [3, 25]. This cell type also shows a constitutive expression of NF– *κ*B [60, 67]. Our third attractor recovers this genetic configuration, showing inactivity of p53, p16, Rb, E2F and ESE–2; and activity of NF– *κ*B, TELase, Snai2, and Cyclin (Fig. 1 c, right).

Cells that have acquired this latter mesenchymal stem–like phenotype have a strong tumorigenic potential, since they show most of the hallmarks of cancer: The low expression of ESE–2 accompanied by the over–activation of Snai2 enable cells to sustain proliferative signals and to evade growth suppressors by undergoing a de–differentiation process [68]. Further, the constitutive activity of Snai2 is associated to an motile and invasive phenotype, and with the avoidance of immune destruction [69] and deregulation of cellular energetics [70]. The de–activation of the senescence markers p16 and p53 and of the tumor suppressor Rb, as well as the over–expression of TELase, confers genome instability that is associated to a mutation–prone phenotype [71] and enable cells to acquire replicative immortality and to resist cell death. Moreover, high levels NF– *κ*B suggest chronic and tumor-promoting cellular inflammation [72].

The correspondence between the recovered attractors in the GRN model simulation and the experimentally observed gene or protein configurations in the studied cellular phenotypes strongly suggests that the proposed core GRN indeed constitutes a regulatory module that comprises a set of components and interactions able to restrict the system to converge to the cellular states observed during spontaneous immortalization. We conclude that the derived core GRN module (Fig. 1 b) includes a set of sufficient and necessary molecular players and interactions that specify epithelial, senescent and stem–like mesenchymal cells.

### Cellular inflammation accelerates the spontaneous immortalization of epithelial cells

Inflammation has been recognized as one of the key drivers of carcinogenesis, partly due to its implication in the EMT [79]. Indeed, several pro-inflammatory cytokines such as TFG- *β* [80] and IL-6 [81], some of which are produced by senescent cells [82], have shown to be strong inducers of EMT. Cells that are exposed to such a pro-inflammatory micro-environment show an over-activation of NF– *κ*B [31, 47], which induces EMT by triggering the expression of mesenchymal markers including Snail [20] and silences the expression of p16 and p53 [83]. To assess whether our model reproduces this increased propensity of de–differentiation into a mesenchymal stem–like phenotype in the presence of cellular inflammation, we calculated the relative size of the basins of attraction of the epithelial, senescent, and mesenchymal stem–like phenotypes (Fig. 1 c), with and without the assumption of a constant over–activation of the NF– *κ*B node in the GRN model. We found that cellular inflammation increases the size of the mesenchymal stem–like attractor from 56.25 to 75% while decreasing the region of convergence of the epithelial (from 17.97 to 6.25%) and of the senescent (from 25.78 to 18.75%) phenotypes (Fig. 1 d). These results are in accordance with the experimental results stated above, in which cellular inflammation is recognized as an important driver of spontaneous immortalization by promoting the induction of de–differentiation of epithelial cells into a mesenchymal stem–like phenotype.

### The proposed GRN reproduces the characteristic phenotypes of 6 different mutant conditions

**Fig. 2:**
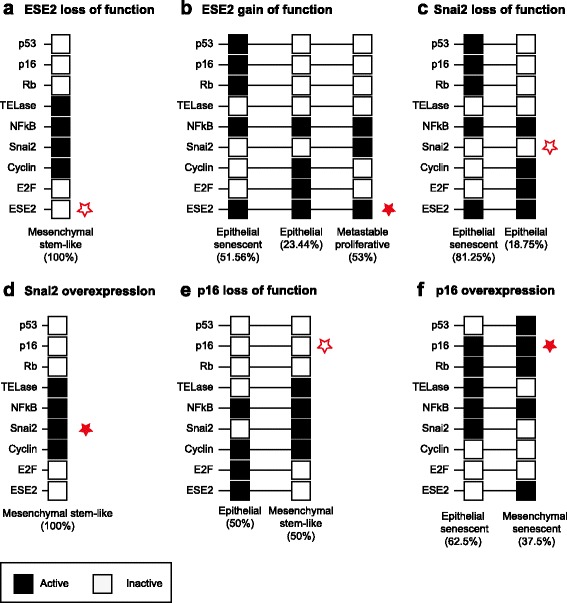
Predicted attractors of loss – and gain of function mutants of the GRN for ESE2 (**a**, **b**), Snai2 (**c**, **d**) and p16 (**e**, **f**). Percent (%) represent the size of the corresponding basin of attraction

To further validate our model, we tested if the GRN (Fig. 1 b) is able to reproduce the phenotypic configurations (in the form of attractors) observed in 6 different mutant conditions. Specifically, we simulated loss– and gain–of–function conditions of ESE–2, Snai2, and p16 (by setting the expression state for the corresponding node constitutively to “1” or “0”, respectively), and compared the resulting attractors to the corresponding gene expression profiles reported in the literature. When simulating *ESE–2 loss–of–function* the model recovers a single attractor, equivalent to a mesenchymal stem–like phenotype with increased Snai2 expression as experimentally observed [18] (Fig. 2 a). Simulations of the *ESE–2 gain of function* mutant results in three attractors that are consistent with the experimental description of the phenotypes resulting from ESE–2 over–expression: an epithelial senescent cell [61], a normal epithelial cell [18], and a EMT intermediate state presenting simultaneous features of both epithelial and mesenchymal states with proliferative phenotype [73] (Fig. 2 b). Simulating *Snai2 loss–of–function* gives rise to two attractors corresponding to normal and senescent epithelial phenotypes, both consistent with experimental observations [18] (Fig. 2 c). Simulating *Snai2 gain–of–function* mutation results in a single attractor corresponding to mesenchymal stem–like phenotype, which is also consistent with experimental observations derived from ectopic over–expression of mesenchymal TFs [74] (Fig. 2 d). These results are also consistent with the TGF- *β*-dependent induction of EMT, which occurs via the activation of Snail by downstream components of the TGF- *β* signaling pathway [75], and which have been successfully reproduced in a recent model of TGF- *β*-driven EMT [76]. *p16 loss–of–function* simulations recover two attractors corresponding to an epithelial and a mesenchymal stem–like cell, which is also consistent with experimental observations [77] (Fig. 2 e). Finally, *gain–of–function simulation of p16* recovered two attractors, one associated with a mesenchymal stem–like but incompletely senescent (due to the lack of p53) phenotype, the other corresponding to an epithelial senescent phenotype. The first prediction is consistent with the immortalization and apoptosis–resistance shown by mesenchymal stem–like cells, as well as with the capability of mesenchymal TFs to abrogate senescence [78]. The second attractor is consistent with the potential for replicative senescence of epithelial cells [56] (Fig. 2 f). In conclusion, we found that our model simulations are consistent with six mutant conditions reported in the literature, providing further validation for our proposed GRN.

### Attractor time–ordered transitions: epigenetic landscape of the uncovered GRN core module

**Fig. 3:**
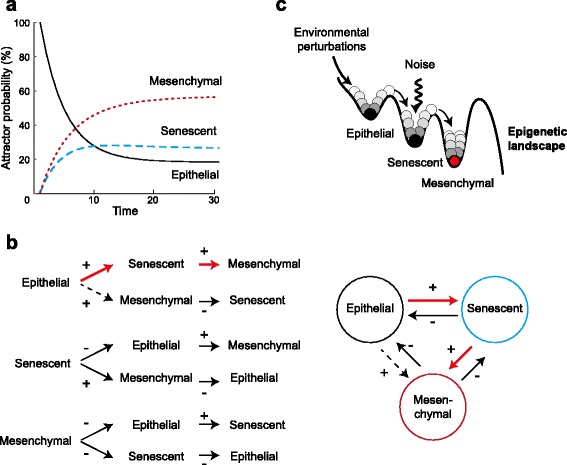
Temporal sequence and global order of cell–fate attainment pattern under the stochastic Boolean GRN model during epithelial carcinogenesis. **a** Maximum probability *p* of attaining each attractor, as a function of time (in iteration steps). The most probable sequence of cell attainment is: epithelial → senescent → mesenchymal stem-like. The error probability used in this case was *ξ*=0.05. The same patterns were obtained with the 3 different error probabilities tested (data not shown). **b** Schematic representation of the possible transitions between pairs of attractors. Arrows indicate the directionality of the transitions. Above each arrow a sign (+) or (−) indicates whether the calculated net transition rate between the corresponding attractors is positive or negative. Red arrows represent the globally consistent ordering for the 3 attractors: the order of the attractors in which all individual transition has a positive net rate, resulting in a global probability flow across the EL. **c** Schematic representation of the time–ordered phenotype transitions along the epigenetic landscape, showing the in–between attractor barrier highs in the landscape

During the tumorigenic transformation of epithelial cells in culture a generic time–ordered series of cell state transitions is observed and can be robustly induced [3]. Normal epithelial cells become first senescent cells, a state which they afterwards overcome to acquire a mesenchymal stem–like phenotype. Interestingly, during the progressive pathological description of epithelial carcinomas in vivo, the temporal pattern with which each of these different cell phenotypes enriches the tissue seems to be tightly ordered and is also generic to all types of such cancers irrespective of the tissue where they first appear. In order to test if the uncovered GRN core module not only underlies and restricts the types of cell phenotypes (attractors) but also their time–ordered transitions, we performed two independent EL: (1) We explored the temporal sequence of attractor attainment, and (2) calculated the consistent global ordering of all the given attractors. To do so, we followed [13, 84] and explored the EL associated to the GRN by implementing a discrete stochastic model as an extension to the deterministic Boolean network model [11] (detailed in Methods). The results of our analysis regarding the temporal sequence of attractor attainment (following the methodology proposed in [13]) show that the most probable temporal order of attractor attainment for a population of cells that initially show and epithelial phenotype correspond to the expected transition from an epithelial to a senescent to a mesenchymal stem–like phenotype (Fig. 3 a). The estimated transition probability matrices are given in Additional file 4: Table S1.

In agreement with these results, following [85] we found a consistent global temporal ordering of the uncovered attractors. This analysis is based on calculating the relative stability of the three different attractors, which is done by computing the Mean First Passage Time (MFPT, shown in Additional file 4: Table S2) between pairs of attractors. These, in turn, epitomize barrier heights in the EL by approximating a measure for the ease of specific transitions. Similar to the previous analysis, the uncovered global ordering of attractors is Epithelial → Senescent → Mesenchymal stem–like (Fig. 3 b). This path corresponds to the only order in which the system can visit the three attractors following a positive net transition rate (Additional file 4: Table S3). These results indicate that, when considering intracellular regulatory constraints alone, the uncovered GRN core module structures the epigenetic landscape in a way that a specific flow across the landscape is preferentially and robustly followed (Fig. 3 c). We anticipate that observed transition rates in vivo are likely to be altered or favored by tissue–level processes and/or additional GRN components underlying epithelial cell sub–differentiation that have not been considered here. These latter restrictions will be modeled in future single cell and multi–level models building up on the framework that has been put forward here.

## Discussion

Multicellularity by definition implies a one–to–many genotype–phenotype map. The genome of a multicellular individual possesses the intrinsic potentiality to implement a developmental process by which all its different cell–types and tissue structures are ultimately established. In the last decades, a coherent theory to explain the development of multicellular organisms as the result of the orchestrating role of GRNs has been developed [8, 10, 11]. The main conclusion is that observable cell states emerge from the self–consistent multi–stable regulatory logic dictated by genome structure and obeyed by (mainly) TFs resulting in stable, steady–states of gene expression. Cancer development and progression is also a phenomenon intrinsic to multicellular organisms. Furthermore, similar to normal development, cancer is robustly established as evidenced by its directionality and phenotypic convergence [2]. Might also cancer be orchestrated by GRN dynamics? Several hypothesis have been presented in this direction such as the cancer attractor theory [2, 6], and the endogenous molecular cellular network hypothesis [86, 87]. In this contribution we adhere to the viewpoint of an intrinsic regulatory network, but focus on a specific developmental process at the cellular level: the robust cell state transitions observed during the tumorigenic transformation of human epithelial cells in culture induced by cellular inflammation and resulting from surpassing a senescent state through EMT – i.e., tumorigenic transformation due to spontaneous immortalization via EMT [63, 88]. We propose that a mechanistic understanding of this process is an important first step to unravel key cellular processes which might be occurring in vivo, where its rate of occurrence is likely to be regulated by tissue–level and systemic conditions directly linked with lifestyle choices, as well as additional regulatory interactions underlying epithelial cell sub–differentiation.

### A generic GRN underlying the epithelial, senescent and mesenchymal stem–like phenotypes

The predominant strategy in the molecular study of cancer and cellular tumorigenic transformation has been to focus on pathways and associated mutations. Aware that signaling pathways are actually embedded in complex regulatory networks, here we assembled curated literature into a GRN comprising the main molecular regulators involved in key cellular processes ubiquitous to carcinogenesis, following a bottom–up approach. Subsequently, we followed a mechanistic approach to address the question of whether we assembled a set of sufficient and necessary molecular players and interactions able to recover the cellular phenotypes and processes documented during the spontaneous immortalization of human epithelial cells in culture. Thus, in this contribution, we analyzed, and validated an experimentally grounded core GRN dynamical model.

Small developmental regulatory modules have been shown to successfully include the necessary and sufficient set of components and interactions for explaining, as manifestations of intrinsic structural and functional constraints imposed by these GRNs, the dynamics of complex processes such as stem cell differentiation [89], cell–fate decision [90] and similar cellular processes during plant morphogenesis [8, 9, 13, 16]. We hypothesized that a similar core developmental module can be formulated in an attempt to explain the cell–fates observed during spontaneous immortalization of human epithelial cells in vitro resulting in a potentially tumorigenic state. In order to show this, we first reduced the proposed larger network (Fig. 1 a) into a regulatory core module, consisting of a small set of key molecular players and the regulatory interactions between them (Fig. 1 b). Although the components of the core module have been previously shown to be involved in EMT, the proposed architecture and topology of this core module had not been proposed before. We extracted from available literature the expression profiles of the generally observable cell states of interest in terms of this minimal set of molecules, and tested whether the reduced GRN, formalized into logical rules, is able to recover the biologically observable expression profiles as stationary and stable network configurations (i.e., attractors). Indeed, we found that the core GRN model converges exactly to the three observed gene expression profiles that correspond to the three phenotypes that *wild type* cells acquire during the process of spontaneous immortalization (Fig. 1 c). Our model analysis also shows that cellular inflammation increases the size of the basin of attraction of the potentially tumorigenic mesenchymal stem–like phenotypic attractor (Fig. 1 d), which is in agreement with the strong tumorigenic effect of inflammation that has been consistently reported in the experimental and clinical literature. Further, our in silico simulations of mutant conditions are also congruent with the corresponding expression profiles (Fig. 2). These results strongly suggest that we have successfully included the key regulators and interactions at play during the establishment of cell steady–states observed during the tumorigenic transformation of human epithelial cells resulting from spontaneous immortalization.

It is noteworthy that our model does not include any hypothetical interaction or component, a common practice in GRN modeling [9, 16, 90]. Our GRN model exclusively integrates available published functional experimental data; indeed, it was a surprising result that the observed dynamical behavior emerged naturally under such conditions. This suggests that despite incomplete information, there is enough molecular data to uncover important restrictions underlying cell behavior during transitions relevant to epithelial carcinogenesis. Consequently, we consider that the networks reported herein may serve as *bona fide* base models useful to integrate novel discoveries, as well as components underlying epithelial cellular sub–differentiation, while following a bottom–up approach in cancer network systems biology.

### Time–ordered transitions of the phenotypic attractors

Discrete GRN models can be used to integrate regulatory mechanisms that not only recapitulate the observed gene expression patterns, but that also reproduce the observed developmental time–ordering of cell phenotypes. This can be done by considering stochasticity in the model in order to explore [11, 17, 84] and/or characterize [85] the associated EL. Importantly, by exploring noise–induced transitions we do not assume that noise alone is the driving force of the transitions, instead, we exploit noise as a tool to explore the GRN–based version of Waddington’s EL and to indirectly characterize its multidimensional structure. Specifically, by calculating the relative stability of the attractors (see Methods) we approximate the in–between attractor barrier heights in the landscape. Furthermore, measures of relative stability can also be exploited to calculate net transition rates measuring the ease of specific inter–attractor transitions and to uncover the predominant developmental path across the epigenetic landscape [91]: ordered transitions sharing positive net transition rates will be preferentially followed. Our results show that such a developmental path follows the time–order of cellular phenotypic states from epithelial, to senescent and finally mesenchymal stem–like cells with a tumorigenic potential (Fig. 3). In other words, the constraints imposed by the GRN structure the associated EL in such a way that an epithelial cell in culture “ball” would naturally roll following such a path, in agreement with the observed spontaneous immortalization process (Fig. 3 c).

It is interesting that the core GRN network is canalized to the few steady–states and the developmental time–ordering consistent with the molecular characterization of cell phenotypes observed during spontaneous immortalization and which correlate with carcinoma progression in vivo. This suggests that specific progressive alterations or particular “abnormal” signaling mechanisms are not necessarily required for a cell to reach a potentially tumorigenic state.

## Conclusions

In this contribution we propose an experimentally grounded GRN model for spontaneous immortalization. For the first time, we integrated a wealth of empirical evidence into a GRN (41 nodes, Fig. 1 a) which we reduced to a core GRN developmental module (9 nodes, Fig. 1 b) that converges to the three phenotypes observed during the spontaneous immortalization of epithelial cells (Fig. 1 c). Simulations of cellular inflammation lead to an increase in the size of the basin of attraction of the potentially tumorigenic mesenchymal stem–like phenotype (Fig. 1 d). Our model also reproduces several experimentally reported mutant conditions (Fig. 2), as well as the time–ordered phenotypic transitions undergone by epithelial cells during the process of spontaneous immortalization (Fig. 3). The proposed GRN constitutes thus a integrative, coherent, and experimentally validated framework which can be used in the future for the integration and analysis of additional signaling and mechanical processes that affect, and are affected by, the oncogenic transformation of the epithelial tissue. To this end, first we will explicitly incorporate into the current model EMT–inducing signaling pathways, (triggered by pro-inflammatory cytokines), increased TFG- *β* concentrations, or changes in the mechanical properties of the surrounding environment. Analysing how these pathways affect the phenotypic outcome, and how different nutritional or pharmacological conditions modulate these trans-differentiation processes (using for example the methodologies proposed in [92, 93]) can aid the design of better prevention and treatment or even oncogenic transformation reversion strategies. To explore this complex interplay between the transcriptional and signaling events driving the phenotypic transitions in individual cells, and the changing properties of the epithelial tissue, we will develop multi-scale tissue level models. Such a framework will enable the systematic assessment of the role on the oncogenic process of several tissue–level constraints such as cell cycle progression, cell–cell interactions, differential proliferation rates, as well as physical fields (e.g., mechanical forces).

Our proposed integrative, quantitative, and dynamical framework contributes to the understanding of the mechanisms underlying the onset and progression of epithelial cancer, which is necessary for devising new and more effective preventive strategies that halt or slow the progression of these diseases.

## Methods

### Construction of the network underlying epithelial carcinogenesis

A total of 159 references, considering both references in the main text and in the Supplementary Information [see Additional file 1: Table S4], were carefully and manually reviewed in order to determine a minimal set of cellular phenotypes and processes which enable a generic representation of spontaneous immortalization (also reported in epithelial carcinogenesis) on the basis of cell state transition events.

Following a bottom–up and an expert knowledge approach we propose a set of cellular dynamical processes ubiquitous to spontaneous immortalization (also reported in epithelial carcinogenesis), namely: replicative cellular senescence EMT driven by inflammation. The cellular phenotypes, epithelial, senescent, and mesenchymal stem–like produced by EMT induction have been largely characterized as biological observables involved in such processes. We take this information as a methodological basis to integrate a generic dynamical network model of spontaneous immortalization. As a first step in network integration, we assembled a set of TFs and additional molecular species involved in the establishment and regulation of these cellular states and processes. Subsequently, we manually retrieved documented regulatory interactions among the molecular species, considering only those supported by experimental evidence. The resulting nodes and regulatory interactions were then assembled manually into the network represented in Fig. 1 a with nodes representing genes and proteins and edges representing activating or inhibitory interactions.

### Network–based gene set enrichment analysis

The bioinformatics tools EnrichNet [94] and TopoGSA [95] were used to perform network–based gene set enrichment analysis and topology–based gene set analysis, respectively. Briefly, EnrichNet maps the input gene set into a molecular interaction network and calculates distances between the genes and pathways/processes in a reference database. TopoGSA also first maps the input gene set into a reference network, to then compute its topological statistics, to finally compare these against the topology of pathways/processes in a reference database. Here a connected human interactome graph extracted from the STRING database, the KEGG and the GO Biological Process databases were used as reference molecular interaction network and database, respectively. Both analysis were performed using the Cytoscape plugin Jepetto [96].

### Network reduction

The regulatory core underlying spontaneous immortalization (Fig. 1 b) was obtained from the large network (Fig. 1 a) by iteratively reducing all the simple mediator (i.e. those with in-degree and out-degree of one) and source (those with no regulators) nodes. This reduction process has been shown to conserve the attractors of the original Boolean network under an asynchronous update method [97]). During the reduction process, we kept the main molecular players controlling cellular senescence (p53 and p16), Cell Cycle (TELase, E2F, Rb and cyclin), differentiation into epithelial cells (ESE-2), differentiation into mesenchymal cells (Snai2), and cellular inflammation (NF- *κ*B), since different activity configurations of these molecular players define the transcriptional identity of the three cellular phenotypes we sought to reproduce with the proposed model.

### Dynamical gene regulatory network model

A Boolean network models a dynamical system assuming both discrete–time and discrete–state variables. This is expressed formally with the mapping:

1 $$ x_{i}(t+1) = F_{i}\left(x_{1}(t),x_{2}(t),\ldots,x_{k}(t)\right),  $$

where the set of functions *F*_*i*_ are logical propositions (or truth tables) expressing the relationship between the genes that share regulatory interactions with the gene *i*, and where the state variables *x*_*i*_(*t*) can take the discrete values 1 or 0 indicating whether the gene *i* is expressed or not at a certain discrete–time *t*, respectively. An experimentally grounded Boolean GRN model is then completely specified by the set of genes proposed to be involved in the process of interest and the associated set of logical functions derived from experimental data [17]. The set of logical functions for the core regulatory module used in this study is given in the Additional file 5. The dynamical analysis of the Boolean network model was conducted using the package *BoolNet* [98] within the R statistical programming environment (www.R-project.org).

### Size of the basins of attraction

The size of the basins of attraction of the GRN corresponds to the percentage of initial Boolean network configurations converging to that attractor, and were calculated for our GRN under *wt* (Fig. 1 d) and under different mutant conditions (Fig. 2) by exhaustively exploring all the 2^9^=512 possible initial configurations of the GRN.

### Epigenetic Landscape characterization

#### Stochastic version of the Boolean GRN

In order to extend the Boolean Network into a discrete stochastic model and then study the properties of its associated EL, the so–called Stochasticity In Nodes model was implemented following [13, 17, 84]. In this model, a constant probability of error *ξ* is introduced for the deterministic Boolean functions. In other words, at each time step, each gene “disobeys” its Boolean function with probability *ξ*. Formally:

2 $$\begin{array}{@{}rcl@{}} & P_{x_{i}(t+1)}\left[F_{i}\left(\mathbf{x}_{reg_{i}}(t)\right)\right] = 1- \xi, \end{array} $$

3 $$\begin{array}{@{}rcl@{}} & P_{x_{i}(t+1)}\left[1 - F_{i}\left(\mathbf{x}_{reg_{i}}(t)\right)\right] = \xi. \end{array} $$

The probability that the value of the now random variable *x*_*i*_(*t*+1) is determined or not by its associated logical function $\phantom {\dot {i}\!}F_{i}(\mathbf {x}_{reg_{i}}(t))$ is 1−*ξ* or *ξ*, respectively.

#### Attractor Transition Probability Estimation

An attractor transition probability matrix *Π* with components:

4 $$ \pi_{ij} = P\left(A_{t+1}=j|A_{t}=i\right),  $$

representing the probability that an attractor *j* is reached from an attractor *i*, was estimated by numerical simulation following [13]. Specifically, for each network state *i* in the state space (2^*n*^) a stochastic one–step transition was simulated a large number of times (≈10,000). The probability of transition from an attractor *i* to an attractor *j* was then estimated as the frequency of times the states belonging to the basin of the attractor *i* were stochastically mapped into a state within the basin of the attractor *j*.

Following the Discrete Time Markov Chains [99] theoretical framework, the estimated transition probability matrix was integrated into a dynamic equation for the probability distribution:

5 $$ P_{A}(t+1) = \Pi P_{A}(t),  $$

where *P*_*A*_(*t*) is the probability distribution over the attractors at time *t*, and *Π* is the transition probability matrix (Additional file 4: Table S1). This equation was then iterated to simulate the temporal evolution of the probability distribution over the attractors starting from a specific initial probability distribution (Fig. 3 a).

#### Attractor relative stability and global ordering analyses

In addition to the calculation of the most probable temporal cell–fate pattern [13], a discrete stochastic GRN model enables the study of the ease for transitioning from one attractor to another [91]. Specifically, a transition barrier in the EL epitomizes the ease for transitioning from one attractor to another. The ease of transitions, in turn, offers a notion of relative stability. It has recently been proposed that the GRN has a consistent global ordering of all cell attractors and intermediate transient states which can be uncovered by measuring the relative stability of all the attractors of a Boolean GRN [85, 91]. Here, the relative stability of each one of the cell states were defined based on the MFPT. Specifically, a relative stability matrix *M* was calculated which reflects the transition barrier between any two states based on the MFPT. Here, the MFPT was numerically estimated. Using the transition probabilities among attractors, a large number sample paths of a finite Markov chain were simulated. The MFPT from attractor *i* to attractor *j* corresponds to the averaged value of the number of steps taken to visit attractor *j* for the fist time, given that the entire probability mass was initially localized at the attractor *i*. The average is taken over the realizations. Following [91], based on the MFPT values a net transition rate between attractor *i* and *j* can be defined as follows:

6 $$ d_{i,j} = \frac{1}{MFPT_{i,j}} - \frac{1}{MFPT_{j,i}}.  $$

This quantity effectively measures the ease of transition as a net probability flow, and is shown for our GRN in Additional file 4: Table S2. For all the calculation involving stochasticity, the robustness of the results was assessed by taking three different values for the probability of error (0.01,0.05,0.1). Stability of the results was assessed by manually changing the number of simulated samples until results become stable.

The consistent global ordering of all attractors uncovered with the core GRN was defined based on the formula proposed in [85]. Briefly, the consistent global ordering of the attractors is given by the attractor permutation in which all transitory net transition rates from an initial attractor to a final attractor are positive. This is schematically represented in Fig. 3 b. Calculated transition probabilities, MFPT, and net transition rate matrices are included in Additional file 4: Table S1, S2, and S3, respectively. R source code implementing all the calculations and analyses is available upon request.

## Additional files

Additional file 1
**Table S4.** Empirical evidence supporting the construction of the large GRN. (PDF 451 kb)

Additional file 2
**Figure S1.** Network -based topological gene set enrichment analysis shows that the topological signature of the molecules in the proposed network is structurally close to the cancer pathways included in the KEGG database. (EPS 39 kb)

Additional file 3 Supplementary Tables. (XLXS 543 kb)

Additional file 4 Supplementary materials. (PDF 69 kb)

Additional file 5 Logic rules and truth tables. (PDF 177 kb)

## Abbreviations

EL: Epigenetic landscape
EMT: Epithelial-Mesenchymal transition
GSEA: Gene set enrichment analysis
GRN: Gene regulatory network
MFPT: Mean first passage time
