# Supplementary material for: Gene regulatory network underlying the immortalization of epithelial cells
Source: BMC Syst Biol. 2017 Feb 16;11:24. doi: 10.1186/s12918-017-0393-5 (PMC5314717; doi:10.1186/s12918-017-0393-5)
Supplement: Additional file 1 — Table S4. Empirical evidence supporting the construction of the large GRN. (PDF 451 kb) [file 12918_2017_393_MOESM1_ESM.pdf]

Table S4: Empirical evidence supporting the construction of the large GRN

Nodes	Edges	Nodes	Description of available experimental data	References
ESE-2	(+)	ESE-2	Elf5 gene itself function as an enhancer for Elf5	Escamilla-Hernandez, R., et al., Genome-wide search identifies Ccnd2 as a direct transcriptional target of Elf5 in mouse mammary gland. BMC Mol Biol, 2010. 11: p. 68.
ESE-2	(+)	ESE-3	Elf5 regulate the expression of ESE-3	Escamilla-Hernandez, R., et al., Genome-wide search identifies Ccnd2 as a direct transcriptional target of Elf5 in mouse mammary gland. BMC Mol Biol, 2010. 11: p. 68.
ESE-1	(+)	ESE-3	ESE-1 up regulates ESE-3 expression	Kas, K., Finger, E., Grall, F., Gu, X., Akbarali, Y., Boltax, J., et al. (2000). ESE-3, a novel member of an epithelium-specific ets transcription factor subfamily, demonstrates different target gene specificity from ESE-1. J Biol Chem, 275(4), 2986-2998.
EGF	(+)	ESE-1	Basal activity of the murine ESX (mESX) promoter is positively regulated by serum stimulation and the EGF-family ligands	Neve, R. M., Parmar, H., Amend, C., Chen, C., Rizzino, A., & Benz, C. C. (2006). Identification of an epithelial-specific enhancer regulating ESX expression. Gene, 367, 118-125.
Her-2/neu	(+)	ESE-1	ESX promoter is a downstream target of the ErbB2 receptor tyrosine kinase	Neve, R. M., Ylstra, B., Chang, C. H., Albertson, D. G., & Benz, C. C. (2002). ErbB2 activation of ESX gene expression. Oncogene, 21(24), 3934-3938.
EGF	(+)	Cyclin	Epidermal growth factor stimulate cyclin D1 gene expression	Scaltriti, M., & Baselga, J. (2006). The epidermal growth factor receptor pathway: a model for targeted therapy. Clin Cancer Res, 12(18), 5268-5272.
ESE-1	(+)	NF-κB	Interaction of ESE1/ELF3 with the NF-κB subunits p65 and p50, acting by enhancing their nuclear translocation and transcriptional activity and by inducing p50 transcription	Longoni, N., Sarti, M., Albino, D., Civenni, G., Malek, A., Ortelli, E., et al. (2013). ETS transcription factor ESE1/ELF3 orchestrates a positive feedback loop that constitutively activates NF-kappaB and drives prostate cancer progression. Cancer Res, 73(14), 4533-4547.
ESE-2	(-)	Slug	Elf5/ESE-2 overexpression was followed by reduced expression of SNAI2 (Slug)	Chakrabarti, R., Hwang, J., Andres Blanco, M., Wei, Y., Lukacisin, M., Romano, R. A., et al. (2012). Elf5 inhibits the epithelial-mesenchymal transition in mammary gland development and breast cancer metastasis by transcriptionally repressing Snail2. Nat Cell Biol, 14(11), 1212-1222.
ESE-2	(-)	Twist	Elf5/ESE-2 overexpression was followed by reduced expression of TWIST1, TWIST2	Chakrabarti, R., Hwang, J., Andres Blanco, M., Wei, Y., Lukacisin, M., Romano, R. A., et al. (2012). Elf5 inhibits the epithelial-mesenchymal transition in mammary gland development and breast cancer metastasis by transcriptionally repressing Snail2. Nat Cell Biol, 14(11), 1212-1222.
Twist	(+)	hTERT	The hypoxia-inducible factor-1α(HIF-1α)-TWIST axis enhance telomerase activity"	Tsai, C. C., Chen, Y. J., Yew, T. L., Chen, L. L., Wang, J. Y., Chiu, C. H., et al. (2011). Hypoxia inhibits senescence and maintains mesenchymal stem cell properties through down-regulation of E2A-p21 by HIF-TWIST. Blood, 117(2), 459-469.
ESE-2	(-)	hTERT	Inhibition of telomerase activity in response to induction of differentiation	Sharma, H. W., Sokoloski, J. A., Perez, J. R., Maltese, J. Y., Sartorelli, A. C., Stein, C. A., et al. (1995). Differentiation of immortal cells inhibits telomerase activity. Proc Natl Acad Sci U S A, 92(26), 12343-12346.
Snai2	(+)	Snai2	Slug is able to activate its own promoter	de Herreros, A. G., Peiro, S., Nassour, M., & Savagner, P. (2010). Snail family regulation and epithelial mesenchymal transitions in breast cancer progression. J Mammary Gland Biol Neoplasia, 15(2), 135-147.
Snail	(+)	Snail	Several reports suggest that Snail can also activate its own synthesis	de Herreros, A. G., Peiro, S., Nassour, M., & Savagner, P. (2010). Snail family regulation and epithelial mesenchymal transitions in breast cancer progression. J Mammary Gland Biol Neoplasia, 15(2), 135-147.
Snail	(+)	Twist	Snail increases the stability of twist	Dave, N., Guaita-Esteruelas, S., Gutarra, S., Frias, A., Beltran, M., Peiro, S., et al. (2011). Functional cooperation between Snail1 and twist in the regulation of ZEB1 expression during epithelial to mesenchymal transition. J Biol Chem, 286(14), 12024-12032.
Snail	(+)	Zeb	Expression of ZEB factors is regulated by snail	Dave, N., Guaita-Esteruelas, S., Gutarra, S., Frias, A., Beltran, M., Peiro, S., et al. (2011). Functional cooperation between Snail1 and twist in the regulation of ZEB1 expression during epithelial to mesenchymal transition. J Biol Chem, 286(14), 12024-12032.
Twist1	(+)	Snai2	Twist1 directly binds to the Slug promoter and activates its transcription	Casas, E., Kim, J., Bendesky, A., Ohno-Machado, L., Wolfe, C. J., & Yang, J. (2011). Snail2 is an essential

				mediator of Twist1-induced epithelial mesenchymal transition and metastasis. Cancer Res, 71(1), 245-254.
Twist1	(+)	Snail	Twist1 regulates the expression of Snail	Leptin, M. (1991). twist and snail as positive and negative regulators during Drosophila mesoderm development. Genes Dev, 5(9), 1568-1576.
Snail	(+)	FOXC2	Overexpression of TGFb, Snail, or Twist increases the expression of FOXC2	Mani, S. A., Yang, J., Brooks, M., Schwaninger, G., Zhou, A., Miura, N., et al. (2007). Mesenchyme Forkhead 1 (FOXC2) plays a key role in metastasis and is associated with aggressive basal-like breast cancers. Proc Natl Acad Sci U S A, 104(24), 10069-10074.
Twist1	(+)	FOXC2	Overexpression of TGFb, Snail, or Twist increases the expression of FOXC2	Mani, S. A., Yang, J., Brooks, M., Schwaninger, G., Zhou, A., Miura, N., et al. (2007). Mesenchyme Forkhead 1 (FOXC2) plays a key role in metastasis and is associated with aggressive basal-like breast cancers. Proc Natl Acad Sci U S A, 104(24), 10069-10074.
Snail	(-)	RKIP	Overexpression of Snail in tumors inhibits RKIP and induce EMT	Wu, K., & Bonavida, B. (2009). The activated NF-kappaB-Snail-RKIP circuitry in cancer regulates both the metastatic cascade and resistance to apoptosis by cytotoxic drugs. Crit Rev Immunol, 29(3), 241-254.
RKIP	(-)	NF-κB	RKIP inhibits NF-kappaB activity through direct interaction with NIK and TAK1	Wu, K., & Bonavida, B. (2009). The activated NF-kappaB-Snail-RKIP circuitry in cancer regulates both the metastatic cascade and resistance to apoptosis by cytotoxic drugs. Crit Rev Immunol, 29(3), 241-254.
Snail	(-)	ESE-2	Snail transcription factors are not present in normal epithelial cells	Sanchez-Tillo, E., Liu, Y., de Barrios, O., Siles, L., Fanlo, L., Cuatrecasas, M., et al. (2012). EMT-activating transcription factors in cancer: beyond EMT and tumor invasiveness. Cell Mol Life Sci, 69(20), 3429-3456.
Snail	(-)	ESE-2	Snail prevents the expression of epithelium-specific genes	Peinado, H., Olmeda, D., & Cano, A. (2007). Snail, Zeb and bHLH factors in tumour progression: an alliance against the epithelial phenotype? Nat Rev Cancer, 7(6), 415-428.
Snail	(-)	Cyclin	Snail regulates components of the early to late G1 transition and the G1/S checkpoint, including the repression of Cyclin D2 transcription and the increase in p21/Cip1"	Vega, S., Morales, A. V., Ocana, O. H., Valdes, F., Fabregat, I., & Nieto, M. A. (2004). Snail blocks the cell cycle and confers resistance to cell death. Genes Dev, 18(10), 1131-1143.
Twist1	(-)	E2F	EMT induction by Twist induce a significant diminution of E2F transcription	Siletz, A., Schnabel, M., Kniazeva, E., Schumacher, A. J., Shin, S., Jeruss, J. S., et al. (2013). Dynamic transcription factor networks in epithelial-mesenchymal transition in breast cancer models. PLoS One, 8(4), e57180.
Twist1	(+)	BMI1	Twist1 and Bmi1 act cooperatively to repress expression of both E-cadherin and p16INK4a	Yang, M. H., Hsu, D. S., Wang, H. W., Wang, H. J., Lan, H. Y., Yang, W. H., et al. (2010). Bmi1 is essential in Twist1-induced epithelial-mesenchymal transition. Nat Cell Biol, 12(10), 982-992.
BMI1	(-)	p16	Twist1 and Bmi1 act cooperatively to repress expression of both E-cadherin and p16INK4a	Yang, M. H., Hsu, D. S., Wang, H. W., Wang, H. J., Lan, H. Y., Yang, W. H., et al. (2010). Bmi1 is essential in Twist1-induced epithelial-mesenchymal transition. Nat Cell Biol, 12(10), 982-992.
Snail	(-)	p53	Snail can suppress p53 by direct binding	Lee, S. H., Lee, S. J., Jung, Y. S., Xu, Y., Kang, H. S., Ha, N. C., et al. (2009). Blocking of p53-Snail binding, promoted by oncogenic K-Ras, recovers p53 expression and function. Neoplasia, 11(1), 22-31, 26p following 31.
Twist1	(-)	p53	Twist over-expression prevents the up regulation of p21CIP1 and p53	Vichalkovski, A., Gresko, E., Hess, D., Restuccia, D. F., & Hemmings, B. A. (2010). PKB/AKT phosphorylation of the transcription factor Twist-1 at Ser42 inhibits p53 activity in response to DNA damage. Oncogene, 29(24), 3554-3565.
TNF-α	(-)	IκB	TNF-α signals phosphorylates inhibitory protein IκB, releasing NF-κB thus activating it	Li, H., & Lin, X. (2008). Positive and negative signaling components involved in TNFalpha-induced NF-kappaB activation. Cytokine, 41(1), 1-8.
IκB	(-)	NF-κB	IκB is the NF-κB inhibitor	Li, H., & Lin, X. (2008). Positive and negative signaling components involved in TNFalpha-induced NF-kappaB activation. Cytokine, 41(1), 1-8.
TGF-β	(+)	TAK1	TGF-β1 treatment induced sequential phosphorylation of TAK1, IKK, IκBα and induced NF-κB gene transcription	Freudlsperger, C., Bian, Y., Contag Wise, S., Burnett, J., Coupar, J., Yang, X., et al. (2013). TGF-beta and NF-kappaB signal pathway cross-talk is mediated through TAK1 and SMAD7 in a subset of head and neck cancers. Oncogene, 32(12), 1549-1559.
TAK1	(-)	IκB	TAK1-mediated phosphorylation of IKKb, resulting in a catalytically active IKK complex	Wang, C., Deng, L., Hong, M., Akkaraju, G. R., Inoue, J.-i., & Chen, Z. J. (2001). TAK1 is a ubiquitin-dependent kinase of MKK and IKK. Nature, 412(6844), 346-351.
IL-6	(-)	IκB	IL-6 stimulation results in a maximal induction of NF-κB activation through phosphorylation of	Wang, L., Walia, B., Evans, J., Gewirtz, A. T., Merlin, D., & Sitaraman, S. V. (2003). IL-6 induces NF-kappa B

		IKKb		activation in the intestinal epithelia. J Immunol, 171(6), 3194-3201.
NF-κB	(+)	TNF-α	NF-κB elements are present in the 5′-flanking regions of several inflammatory response genes, including the cytokines and regulate positive cytokine induction	Cloutier, A., T. Ear, E. Blais-Charron, C. M. Dubois, and P. P. McDonald. 2007. Differential involvement of NF-kappaB and MAP kinase pathways in the generation of inflammatory cytokines by human neutrophils. J. Leukoc. Biol. 81: 567–577.
NF-κB	(+)	TGF-β	NF-κB elements are present in the 5′-flanking regions of several inflammatory response genes, including the cytokines and regulate positive cytokine induction	Hiscott, J., et al., Characterization of a functional NF-kappa B site in the human interleukin 1 beta promoter: evidence for a positive autoregulatory loop. Mol Cell Biol, 1993. 13(10): p. 6231-40
NF-κB	(+)	IL-6	NF-κB elements are present in the 5′-flanking regions of several inflammatory response genes, including the cytokines and regulate positive cytokine induction	Cloutier, A., T. Ear, E. Blais-Charron, C. M. Dubois, and P. P. McDonald. 2007. Differential involvement of NF-kappaB and MAP kinase pathways in the generation of inflammatory cytokines by human neutrophils. J. Leukoc. Biol. 81: 567–577.
NF-κB	(+)	ESE-1	Nuclear factor κB-mediated induction of the Ets transcription factor ESE-1	Grall, F., et al., Responses to the proinflammatory cytokines interleukin-1 and tumor necrosis factor alpha in cells derived from rheumatoid synovium and other joint tissues involve nuclear factor kappaB-mediated induction of the Ets transcription factor ESE-1. Arthritis Rheum, 2003. 48(5): p. 1249-60.
NF-κB	(+)	ESE-3	Nuclear factor κB-mediated induction of the Ets transcription factor ESE-3	Wu, J., et al., Regulation of epithelium-specific Ets-like factors ESE-1 and ESE-3 in airway epithelial cells: potential roles in airway inflammation. Cell Res, 2008. 18(6): p. 649-63.
NF-κB	(+)	Snai2	SLUG is activated by nuclear factor kappa B	Wang, Y., Yue, B., Yu, X., Wang, Z., & Wang, M. (2013). SLUG is activated by nuclear factor kappa B and confers human alveolar epithelial A549 cells resistance to tumor necrosis factor-alpha-induced apoptosis. World J Surg Oncol, 11, 12.
NF-κB	(+)	Snail	Snail can be stabilized by TNF-α through the activation of the NF-κB	Wu, Y., Deng, J., Rychahou, P. G., Qiu, S., Evers, B. M., & Zhou, B. P. (2009). Stabilization of snail by NF-κB is required for inflammation-induced cell migration and invasion. Cancer Cell, 15(5), 416-428.
NF-κB	(+)	Zeb	Nuclear factor kappa B (NF-κB), in transient transfection assays of subunit p65 increased ZEB-1 promoter activity	Chua, H.L., et al., NF-kappaB represses E-cadherin expression and enhances epithelial to mesenchymal transition of mammary epithelial cells: potential involvement of ZEB-1 and ZEB-2. Oncogene, 2007. 26(5): p. 711-24
NF-κB	(+)	Twist	NF-κB-mediated transcriptional up regulation of Twist1	Li, C.W., et al., Epithelial-mesenchymal transition induced by TNF-alpha requires NF-kappaB-mediated transcriptional upregulation of Twist1. Cancer Res, 2012. 72(5): p. 1290-300.
NF-κB	(+)	FOXC2	A number of novel NF-κB/IKK-dependent genes were identified including FOXC2<br>Unequivocal evidence shows that NF-κB activation has been associated with the induction of many transcription factors involved in EMT, such as Snail, Slug, Twist, and ZEB1/ZEB2	Li, X., et al., IKKα, IKKβ, and NEMO/IKKγ are each required for the NF-κB-mediated inflammatory response program. Journal of Biological Chemistry, 2002. 277(47): p. 45129-45140<br>Min, C., Eddy, S. F., Sherr, D. H., & Sonenshein, G. E. (2008). NF-κB and epithelial to mesenchymal transition of cancer. Journal of cellular biochemistry, 104(3), 733-744.
NF-κB	(+)	Cyclin	NF-kappaB activates cyclin D1 expression	Guttridge, D.C., et al., NF-kappaB controls cell growth and differentiation through transcriptional regulation of cyclin D1. Mol Cell Biol, 1999. 19(8): p. 5785-99.
NF-κB	(-)	p53	the complete or partial repression of p53 observed in many tumors can be the result of constitutive activation of NF-kappaB	Gurova, K.V., et al., Small molecules that reactivate p53 in renal cell carcinoma reveal a NF-kappaB-dependent mechanism of p53 suppression in tumors. Proc Natl Acad Sci U S A, 2005. 102(48): p. 17448-53
p16	(-)	CBX7	p16 directly repress expression of Polycomb group (PcG) proteins CBX7	Overhoff, M. G., Garbe, J. C., Koh, J., Stampfer, M. R., Beach, D. H., & Bishop, C. L. (2014). Cellular senescence mediated by p16INK4A-coupled miRNA pathways. Nucleic Acids Res, 42(3), 1606-1618.
p16	(-)	EED	p16 directly repress expression of Polycomb group (PcG) proteins EED	Overhoff, M. G., Garbe, J. C., Koh, J., Stampfer, M. R., Beach, D. H., & Bishop, C. L. (2014). Cellular senescence mediated by p16INK4A-coupled miRNA pathways. Nucleic Acids Res, 42(3), 1606-1618.
p16	(-)	EZH2	p16 directly repress expression of Polycomb group (PcG) proteins EZH2	Overhoff, M. G., Garbe, J. C., Koh, J., Stampfer, M. R., Beach, D. H., & Bishop, C. L. (2014). Cellular senescence mediated by p16INK4A-coupled miRNA pathways. Nucleic Acids Res, 42(3), 1606-1618.
p16	(-)	Suz12	p16 directly repress expression of Polycomb	Overhoff, M. G., Garbe, J. C., Koh, J., Stampfer, M. R.,

			group (PcG) proteins Suz12	Beach, D. H., & Bishop, C. L. (2014). Cellular senescence mediated by p16INK4A-coupled miRNA pathways. Nucleic Acids Res, 42(3), 1606-1618.
p16	(-)	BMI1	In the absence of Bmi1 p16Ink4a expresses	Guo, W.J., et al., Mel-18, a polycomb group protein, regulates cell proliferation and senescence via transcriptional repression of Bmi-1 and c-Myc oncoproteins. Mol Biol Cell, 2007. 18(2): p. 536-46.
CBX7	(-)	p16	CBX7 repress the Ink4a/Arf locus	Bernard, D., Martinez-Leal, J. F., Rizzo, S., Martinez, D., Hudson, D., Visakorpi, T., et al. (2005). CBX7 controls the growth of normal and tumor-derived prostate cells by repressing the Ink4a/Arf locus. Oncogene, 24(36), 5543-5551.
EED	(-)	p16	The ability to repress the INK4A-ARF locus requires EZH2-containing Polycomb-Repressive Complex 2 (PRC2) association	Bracken, A. P., Kleine-Kohlbrecher, D., Dietrich, N., Pasini, D., Gargiulo, G., Beekman, C., et al. (2007). The Polycomb group proteins bind throughout the INK4A-ARF locus and are disassociated in senescent cells. Genes & development, 21(5), 525-530.
EZH2	(-)	p16	The ability to repress the INK4A-ARF locus requires EZH2-containing Polycomb-Repressive Complex 2 (PRC2) association	Bracken, A. P., Kleine-Kohlbrecher, D., Dietrich, N., Pasini, D., Gargiulo, G., Beekman, C., et al. (2007). The Polycomb group proteins bind throughout the INK4A-ARF locus and are disassociated in senescent cells. Genes & development, 21(5), 525-530.
Suz12	(-)	p16	The ability to repress the INK4A-ARF locus requires EZH2-containing Polycomb-Repressive Complex 2 (PRC2) association	Bracken, A. P., Kleine-Kohlbrecher, D., Dietrich, N., Pasini, D., Gargiulo, G., Beekman, C., et al. (2007). The Polycomb group proteins bind throughout the INK4A-ARF locus and are disassociated in senescent cells. Genes & development, 21(5), 525-530.
BMI1	(-)	p16	Bmi1 negatively regulates Ink4a	Guo, W.J., et al., Mel-18, a polycomb group protein, regulates cell proliferation and senescence via transcriptional repression of Bmi-1 and c-Myc oncoproteins. Mol Biol Cell, 2007. 18(2): p. 536-46.
p16	(-)	CDK2	Binding of p16 inhibits the cyclin-dependent kinase 2	McConnell, B.B., et al., Induced expression of p16(INK4a) inhibits both CDK4- and CDK2-associated kinase activity by reassortment of cyclin-CDK-inhibitor complexes. Mol Cell Biol, 1999. 19(3): p. 1981-9.
p16	(-)	CDK4	Binding of p16 inhibits the cyclin-dependent kinase 4	McConnell, B.B., et al., Induced expression of p16(INK4a) inhibits both CDK4- and CDK2-associated kinase activity by reassortment of cyclin-CDK-inhibitor complexes. Mol Cell Biol, 1999. 19(3): p. 1981-9.
p16	(-)	CDK6	The p16 (INK4a) tumor suppressor binds to Cdk4/6	Villacanas, O., J.J. Perez, and J. Rubio-Martinez, Structural analysis of the inhibition of Cdk4 and Cdk6 by p16(INK4a) through molecular dynamics simulations. J Biomol Struct Dyn, 2002. 20(3): p. 347-58.
CDK2	(-)	Rb	Cyclin-dependent kinase 2-4/cyclin D complex inhibits the phosphorylation of pRb family proteins	McConnell, B.B., et al., Induced expression of p16(INK4a) inhibits both CDK4- and CDK2-associated kinase activity by reassortment of cyclin-CDK-inhibitor complexes. Mol Cell Biol, 1999. 19(3): p. 1981-9.
CDK4	(-)	Rb	Cyclin-dependent kinase 2-4/cyclin D complex inhibits the phosphorylation of pRb family proteins	McConnell, B.B., et al., Induced expression of p16(INK4a) inhibits both CDK4- and CDK2-associated kinase activity by reassortment of cyclin-CDK-inhibitor complexes. Mol Cell Biol, 1999. 19(3): p. 1981-9.
CDK6	(-)	Rb	Cdk6 inhibits phosphorylation of the retinoblastoma protein	Villacanas, O., J.J. Perez, and J. Rubio-Martinez, Structural analysis of the inhibition of Cdk4 and Cdk6 by p16(INK4a) through molecular dynamics simulations. J Biomol Struct Dyn, 2002. 20(3): p. 347-58.
p16	(+)	NF-κB	Through a proteomics analysis of senescent chromatin, we identified the nuclear factor-kappaB (NF-κB) subunit p65 as a major transcription factor that accumulates on chromatin of senescent cells	Chien, Y., et al., Control of the senescence-associated secretory phenotype by NF-kappaB promotes senescence and enhances chemosensitivity. Genes Dev, 2011. 25(20): p. 2125-36.
p14	(-)	MDM2	p14 inactivates MDM2	Lowe, S.W. and C.J. Sherr, Tumor suppression by Ink4a-Arf: progress and puzzles. Curr Opin Genet Dev, 2003. 13(1): p. 77-83.
MDM2	(-)	p53	Mdm2 promotes the rapid degradation of p53	Haupt, Y., Maya, R., Kazaz, A., & Oren, M. (1997). Mdm2 promotes the rapid degradation of p53. Nature, 387(6630), 296-299.
Cyclin	(+)	E2F	Cyclin E does function as an E2F activator	Johnson, D.G., K. Ohtani, and J.R. Nevins, Autoregulatory control of E2F1 expression in response to positive and negative regulators of cell cycle progression. Genes Dev, 1994. 8(13): p. 1514-25.
Rb	(-)	E2F	Rb protein associate with transcription factor E2F1, localizing it to the cytoplasm and thus	Kato, J., et al., Direct binding of cyclin D to the retinoblastoma gene product (pRb) and pRb

			preventing transcription of E2F1 target genes	phosphorylation by the cyclin D-dependent kinase CDK4. Genes Dev, 1993. 7(3): p. 331-42
Cyclin	(-)	Rb	Rb function, including the ability to interact with E2F, was regulated by phosphorylation and that the primary kinase responsible was the D-type cyclin-dependent kinases	Kato, J., et al., Direct binding of cyclin D to the retinoblastoma gene product (pRb) and pRb phosphorylation by the cyclin D-dependent kinase CDK4. Genes Dev, 1993. 7(3): p. 331-42
E2F	(+)	Cyclin	E2F also directs the synthesis of both cyclin E and cdk2, creating the kinase activity responsible for activation of replication.	Johnson, D. G., Ohtani, K., & Nevins, J. R. (1994). Autoregulatory control of E2F1 expression in response to positive and negative regulators of cell cycle progression. Genes Dev, 8(13), 1514-1525.
p53	(+)	p21	Induction of p21 by p53 following DNA	He, G., Siddik, Z. H., Huang, Z., Wang, R., Koomen, J., Kobayashi, R., et al. (2005). Induction of p21 by p53 following DNA damage inhibits both Cdk4 and Cdk2 activities. Oncogene, 24(18), 2929-2943.
p21	(-)	CDK2	p21 inhibits both Cdk4 and Cdk2 activities	He, G., Siddik, Z. H., Huang, Z., Wang, R., Koomen, J., Kobayashi, R., et al. (2005). Induction of p21 by p53 following DNA damage inhibits both Cdk4 and Cdk2 activities. Oncogene, 24(18), 2929-2943.
p21	(-)	CDK4	p21 inhibits both Cdk4 and Cdk2 activities	He, G., Siddik, Z. H., Huang, Z., Wang, R., Koomen, J., Kobayashi, R., et al. (2005). Induction of p21 by p53 following DNA damage inhibits both Cdk4 and Cdk2 activities. Oncogene, 24(18), 2929-2943.
Telun	(+)	ATM	ATM is a primary mediator for uncapped telomeres	Herbig, U., et al., Telomere shortening triggers senescence of human cells through a pathway involving ATM, p53, and p21(CIP1), but not p16(INK4a). Mol Cell, 2004. 14(4): p. 501-13.
Telun	(+)	ATR	ATR substitutes the role of ATM to activate p53 through Chk1-induced p53 phosphorylation	Herbig, U., et al., Telomere shortening triggers senescence of human cells through a pathway involving ATM, p53, and p21(CIP1), but not p16(INK4a). Mol Cell, 2004. 14(4): p. 501-13.
ATM	(-)	MDM2	Phosphorylation of MDM2 by ATM attenuates the capability of MDM2 in exporting nuclear p53 to cytoplasm for subsequent degradation, thereby enabling p53 accumulation	Maya, R., et al., ATM-dependent phosphorylation of Mdm2 on serine 395: role in p53 activation by DNA damage. Genes Dev, 2001. 15(9): p. 1067-77.
ATM	(+)	Chk2	ATM is required for Chk2 activation	Smith, J., Tho, L. M., Xu, N., & Gillespie, D. A. (2010). The ATM-Chk2 and ATR-Chk1 pathways in DNA damage signaling and cancer. Advances in cancer research(108), 73-112.
Chk2	(+)	p53	Chk2 phosphorylates and activates p53	Jack, M. T., Woo, R. A., Hirao, A., Cheung, A., Mak, T. W., & Lee, P. W. (2002). Chk2 is dispensable for p53-mediated G1 arrest but is required for a latent p53-mediated apoptotic response. Proceedings of the National Academy of Sciences, 99(15), 9825-9829.
ATR	(+)	Chk1	ATRis required for Chk1 activation	Smith, J., Tho, L. M., Xu, N., & Gillespie, D. A. (2010). The ATM-Chk2 and ATR-Chk1 pathways in DNA damage signaling and cancer. Advances in cancer research(108), 73-112.
p53	(+)	PML	The p53 protein activate PML	de Stanchina, E., et al., PML is a direct p53 target that modulates p53 effector functions. Mol Cell, 2004. 13(4): p. 523-35.
PML	(-)	MDM2	PML forms a positive feedback loop with p53 to trigger cellular senescence by inhibiting p53 degradation by MDM2	Bernardi, R., et al., PML regulates p53 stability by sequestering Mdm2 to the nucleolus. Nat Cell Biol, 2004. 6(7): p. 665-72.
PML	(+)	p16	PML recruits p16 leading to senescence	Vernier, M., et al., Regulation of E2Fs and senescence by PML nuclear bodies. Genes Dev, 2011. 25(1): p. 41-50.
E2F	(+)	C-MYC	E2F sites are also found in the promoters of several cellular proto-oncogenes, including c-myc	DeGregori, J., T. Kowalik, and J.R. Nevins, Cellular targets for activation by the E2F1 transcription factor include DNA synthesis- and G1/S-regulatory genes. Mol Cell Biol, 1995. 15(8): p. 4215-24.
C-MYC	(+)	BMI1	Bmi-1 is a c-Myc target	Mel-18, a polycomb group protein, regulates cell proliferation and senescence via transcriptional repression of Bmi-1 and c-Myc oncoproteins
E2F	(+)	SUZ12	We have previously identified SUZ12 as an E2F target gene	Kirmizis, A., S.M. Bartley, and P.J. Farnham, Identification of the polycomb group protein SU(Z)12 as a potential molecular target for human cancer therapy. Mol Cancer Ther, 2003. 2(1): p. 113-21
E2F	(+)	EZH2	EZH2 is downstream of the pRB–E2F pathway	Bracken, A. P., Kleine-Kohlbrecher, D., Dietrich, N., Pasini, D., Gargiulo, G., Beekman, C., et al. (2007). The Polycomb group proteins bind throughout the INK4A-ARF locus and are disassociated in senescent cells. Genes & development, 21(5), 525-530.
Cyclin	(+)	CDK2	The active site of cyclin-dependent kinases is	Liu, J., & Kipreos, E. T. (2000). Evolution of cyclin-

			blocked in the absence of cyclin	dependent kinases (CDKs) and CDK-activating kinases (CAKs): differential conservation of CAKs in yeast and metazoa. Molecular Biology and Evolution, 17(7), 1061-1074.
Cyclin	(+)	CDK4	The active site of cyclin-dependent kinases is blocked in the absence of cyclin	Liu, J., & Kipreos, E. T. (2000). Evolution of cyclin-dependent kinases (CDKs) and CDK-activating kinases (CAKs): differential conservation of CAKs in yeast and metazoa. Molecular Biology and Evolution, 17(7), 1061-1074.
Cyclin	(+)	CDK6	The active site of cyclin-dependent kinases is blocked in the absence of cyclin	Liu, J., & Kipreos, E. T. (2000). Evolution of cyclin-dependent kinases (CDKs) and CDK-activating kinases (CAKs): differential conservation of CAKs in yeast and metazoa. Molecular Biology and Evolution, 17(7), 1061-1074.
hTERT	(-)	Telun	The enzyme telomerase, add the telomeric sequence to chromosome ends de novo	Liu, Z., et al., Telomerase reverse transcriptase promotes epithelial-mesenchymal transition and stem cell-like traits in cancer cells. Oncogene, 2013. 32(36): p. 4203-13.
hTERT	(-)	p53	Human telomerase catalytic subunit (hTERT) suppresses p53	Jin, X., Beck, S., Sohn, Y.-W., Kim, J.-K., Kim, S.-H., Yin, J., et al. (2010). Human telomerase catalytic subunit (hTERT) suppresses p53-mediated anti-apoptotic response via induction of basic fibroblast growth factor. Experimental & molecular medicine, 42(8), 574-582.
hTERT	(-)	p16	Telomerase expression prevents replicative senescence.<br>Downregulation of p16 activity maybe promoted by the introduction of exogenous hTERT	Choi, D., Whittier, P. S., Oshima, J., & Funk, W. D. (2001). Telomerase expression prevents replicative senescence but does not fully reset mRNA expression patterns in Werner syndrome cell strains. *FASEB J*, *15*(6), 1014-1020<br>Egbuniwe, O., Idowu, B. D., Funes, J. M., Grant, A. D., Renton, T., & Di Silvio, L. (2011). P16/p53 expression and telomerase activity in immortalized human dental pulp cells. *Cell Cycle*, *10*(22), 3912-3919.
