# Supplementary material for: Gene regulatory network underlying the immortalization of epithelial cells
Source: BMC Syst Biol. 2017 Feb 16;11:24. doi: 10.1186/s12918-017-0393-5 (PMC5314717; doi:10.1186/s12918-017-0393-5)
Supplement: Additional file 4 — Supplementary materials. (PDF 69 kb) [file 12918_2017_393_MOESM4_ESM.pdf]

Supplementary material

Gene regulatory network underlying the immortalization of epithelial cells

Luis Fernando Méndez-López, Jose Davila-Velderrain, Elisa Domínguez-Hüttinger, Christian, Enríquez-Olguín, Juan Carlos Martínez-García, and Elena R Alvarez-Buylla *

*eabuylla@gmail.com

Additional tables

Table S1: Transition probability matrices

**Attractors**	Epithelial	Senescent	Mesenchymal stem–like
Noise=0.01			
Epithelial	0.9635	0.0146	0.0219
Senescent	0.0121	0.9735	0.0145
Mesenchymal stem-like	0.0061	0.0070	0.9869
Noise=0.05			
Epithelial	0.9635	0.01461	0.02192
Senescent	0.0121	0.9735	0.0145
Mesenchymal stem-like	0.0061	0.0070	0.9869
Noise=0.1			
Epithelial	0.6692	0.1383	0.1925
Senescent	0.0908	0.7675	0.1417
Mesenchymal stem-like	0.0573	0.06539	0.8773

Additional text– Robustness analysis

We further validated the dynamical GRN model by testing its robustness to perturbations of the logical rules. Specifically, we tested the robustness of the predicted attractors by generating a set of 10,000 perturbed networks, calculating their respective attractors, and then counting the occurrences of the original attractors within the perturbed set. We generated each perturbed network by choosing a function of the

Table S2: Mean First Passage Time Matrices

**Attractors**	Epithelial	Senescent	Mesenchymal stem-like
Noise=0.01			
Epithelial	0	101.7128	51.8735
Senescent	111.9410	0	61.2607
M	136.1419	123.9060	0
Noise=0.05			
Epithelial	0	20.9902	11.79566
Senescent	23.9068	0	13.9003
Mesenchymal stem-like	28.2412	27.51117	0
Noise=0.1			
Epithelial	0	10.6632	5.76263
Senescent	13.73	0	6.5515
Mesenchymal stem-like	15.4678	13.1307	0

network at random and flipping a single bit in this function [1]. We performed four complementary *in silico* based experiments following this general robustness analysis. First, we estimated the fraction of occurrences of the three original attractors (*i.e.*, their robustness). Then, we repeated the experiment three times, but each time estimating the robustness of each individual attractor. For these four experiments we estimated a robustness (*i.e.*, fraction of times) of 0.7439, 0.905, 0.923, and 0.902, respectively. Hence, out of 10,000 random networks generated by *in silico* perturbations to the logical rules, a major fraction recovered the original attractors; as it is expected for a developmental (core) regulatory module that is robust both to transient (initial) and genetic perturbations [2]. This result supports the view that the core GRN uncovered here is indeed a regulatory network module driving developmental dynamics.

Table S3: Net transition rate matrices

**Attractors**	Epithelial	Senescent	Mesenchymal stem–like
Noise=0.01			
Epithelial	0	0.0009	0.0119
Senescent	-0.0009	0	0.0083
Mesenchymal stem-like	-0.0119	-0.0083	0
Noise=0.05			
Epithelial	0	0.0009	0.0119
Senescent	-0.0009	0	0.0083
Mesenchymal stem–like	-0.0119	-0.0083	0
Noise=0.1			
Epithelial	0	0.02095	0.1089
Senescent	-0.02095	0	0.07648
Mesenchymal stem–like	-0.1089	-0.0765	0

It also constitutes a mechanistic explanation (for definitions, see [3]) to the generic cell phenotypes observed during spontaneous immortalization *in vitro* and which correlate with the cellular description of carcinoma progression *in vivo*.

References

[1] Müssel, C., Hopfensitz, M., Kestler, H.A.: BoolNet—an R package for generation, reconstruction and analysis of Boolean networks. Bioinformatics **26**(10), 1378–1380 (2010)

[2] Espinosa-Soto, C., Padilla-Longoria, P., Alvarez-Buylla, E.R.: A gene regulatory network model for cell-fate determination during Arabidopsis thaliana flower development that is robust and recovers experimental gene expression profiles. Plant Cell Online **16**(11), 2923–2939 (2004)

[3] Davila-Velderrain, J., Martinez-Garcia, J.C., Alvarez-Buylla, E.R.: Descriptive vs. Mechanistic Network Models in Plant Development in the Post-Genomic Era. Plant Funct. Genomics Methods Protoc., 455–479 (2015)
