# Supplementary material for: Gene regulatory network underlying the immortalization of epithelial cells
Source: BMC Syst Biol. 2017 Feb 16;11:24. doi: 10.1186/s12918-017-0393-5 (PMC5314717; doi:10.1186/s12918-017-0393-5)
Supplement: Additional file 5 — Logic rules and truth tables. (PDF 177 kb) [file 12918_2017_393_MOESM5_ESM.pdf]

Supplementary Text 1: Logic Rules and Truth Tables

Mesenchymal phenotype:

- **Snai2**= (!ESE2 & !NFkB & !Snai2) | (!ESE2 & !NFkB & Snai2) | (!ESE2 & NFkB & !Snai2) | (!ESE2 & NFkB & Snai2) | (ESE2 & NFkB & Snai2)

ESE2	NF-kB	Snai2	**Snai2**
0	0	0	**1**
0	0	1	**1**
0	1	0	**1**
0	1	1	**1**
1	0	0	**0**
1	0	1	**0**
1	1	0	**0**
1	1	1	**1**

Epithelial phenotype:

- **ESE2**=(!NFkB & !Snai2 & !ESE2) | (!NFkB & !Snai2 & ESE2) | (!NFkB & Snai2 & ESE2) | (NFkB & !Snai2 & !ESE2) | (NFkB & !Snai2 & ESE2)

NF-kB	Snai2	ESE2	**ESE2**
0	0	0	**1**
0	0	1	**1**
0	1	0	**0**
0	1	1	**1**
1	0	0	**1**
1	0	1	**1**
1	1	0	**0**
1	1	1	**0**

Cellular inflammation:

- **NF-kB**= !(!ESE2 & !p16 & !Snai2 & !NFkB)

ESE2	p16	Snai2	NF-kB	**NF-kB**
0	0	0	0	**0**
0	0	0	1	**1**
0	0	1	0	**1**
0	0	1	1	**1**
0	1	0	0	**1**
0	1	0	1	**1**
0	1	1	0	**1**
0	1	1	1	**1**
1	0	0	0	**1**

1	0	0	1	**1**
1	0	1	0	**1**
1	0	1	1	**1**
1	1	0	0	**1**
1	1	0	1	**1**
1	1	1	0	**1**
1	1	1	1	**1**

Cellular senescence:

- p16= (!p16 & !E2F & p53 & !TELasa & !Snai2) | (!p16 & !E2F & p53 & !TELasa & Snai2) | (!p16 & !E2F & p53 & TELasa & !Snai2) | (!p16 & E2F & p53 & !TELasa & !Snai2) | (!p16 & E2F & p53 & !TELasa & Snai2) | (!p16 & E2F & p53 & TELasa & !Snai2) | (p16 & !E2F & !p53 & !TELasa & !Snai2) | (p16 & !E2F & p53 & !TELasa & !Snai2) | (p16 & !E2F & p53 & !TELasa & Snai2) | (p16 & !E2F & p53 & TELasa & !Snai2) | (p16 & E2F & !p53 & !TELasa & !Snai2) | (p16 & E2F & !p53 & !TELasa & Snai2) | (p16 & E2F & !p53 & TELasa & !Snai2) | (p16 & E2F & !p53 & TELasa & Snai2) | (p16 & E2F & p53 & !TELasa & !Snai2) | (p16 & E2F & p53 & !TELasa & Snai2) | (p16 & E2F & p53 & TELasa & !Snai2) | (p16 & E2F & p53 & TELasa & Snai2) | (p16 & !E2F & !p53 & TELasa & !Snai2)

P16	E2F	P53	TELasa	Snai2	**P16**
0	0	0	0	0	**0**
0	0	0	0	1	**0**
0	0	0	1	0	**0**
0	0	0	1	1	**0**
0	0	1	0	0	**1**
0	0	1	0	1	**1**
0	0	1	1	0	**1**
0	0	1	1	1	**0**
0	1	0	0	0	**0**
0	1	0	0	1	**0**
0	1	0	1	0	**0**
0	1	0	1	1	**0**
0	1	1	0	0	**1**
0	1	1	0	1	**1**
0	1	1	1	0	**1**
0	1	1	1	1	**0**
1	0	0	0	0	**1**
1	0	0	0	1	**0**
1	0	0	1	0	**1**
1	0	0	1	1	**0**
1	0	1	0	0	**1**
1	0	1	0	1	**1**
1	0	1	1	0	**1**
1	0	1	1	1	**0**

1	1	0	0	0	**1**
1	1	0	0	1	**1**
1	1	0	1	0	**1**
1	1	0	1	1	**1**
1	1	1	0	0	**1**
1	1	1	0	1	**1**
1	1	1	1	0	**1**
1	1	1	1	1	**1**

- **p53=** (!p53 & !NFkB & !TELasa & !p16 & !Snai2) | (!p53 & !NFkB & !TELasa & p16 & !Snai2) | (!p53 & NFkB & !TELasa & p16 & !Snai2) | (p53 & !NFkB & !TELasa & !p16 & !Snai2) | (p53 & !NFkB & !TELasa & p16 & !Snai2) | (p53 & NFkB & !TELasa & p16 & !Snai2)

p53	NF-kB	TELasa	p16	Snai2	**p53**
0	0	0	0	0	**1**
0	0	0	0	1	**0**
0	0	0	1	0	**1**
0	0	0	1	1	**0**
0	0	1	0	0	**0**
0	0	1	0	1	**0**
0	0	1	1	0	**0**
0	0	1	1	1	**0**
0	1	0	0	0	**0**
0	1	0	0	1	**0**
0	1	0	1	0	**1**
0	1	0	1	1	**0**
0	1	1	0	0	**0**
0	1	1	0	1	**0**
0	1	1	1	0	**0**
0	1	1	1	1	**0**
1	0	0	0	0	**1**
1	0	0	0	1	**0**
1	0	0	1	0	**1**
1	0	0	1	1	**0**
1	0	1	0	0	**0**
1	0	1	0	1	**0**
1	0	1	1	0	**0**
1	0	1	1	1	**0**
1	1	0	0	0	**0**
1	1	0	0	1	**0**
1	1	0	1	0	**1**
1	1	0	1	1	**0**
1	1	1	0	0	**0**
1	1	1	0	1	**0**

1	1	1	1	0	**0**
1	1	1	1	1	**0**

Cell cycle:

- **Cyclin**= (!ESE2 & !E2F & !p16 & !NFkB & !Snai2) | (!ESE2 & !E2F & !p16 & NFkB & !Snai2) | (!ESE2 & !E2F & !p16 & NFkB & Snai2) | (!ESE2 & E2F & !p16 & !NFkB & !Snai2) | (!ESE2 & E2F & !p16 & NFkB & !Snai2) | (!ESE2 & E2F & !p16 & NFkB & Snai2) | (ESE2 & !E2F & !p16 & !NFkB & !Snai2) | (ESE2 & !E2F & !p16 & NFkB & !Snai2) | (ESE2 & E2F & !p16 & !NFkB & !Snai2) | (ESE2 & E2F & !p16 & NFkB & !Snai2)

ESE2	E2F	p16	NF-kB	Snai2	**Cyclin**
0	0	0	0	0	**1**
0	0	0	0	1	**0**
0	0	0	1	0	**1**
0	0	0	1	1	**1**
0	0	1	0	0	**0**
0	0	1	0	1	**0**
0	0	1	1	0	**0**
0	0	1	1	1	**0**
0	1	0	0	0	**1**
0	1	0	0	1	**0**
0	1	0	1	0	**1**
0	1	0	1	1	**1**
0	1	1	0	0	**0**
0	1	1	0	1	**0**
0	1	1	1	0	**0**
0	1	1	1	1	**0**
1	0	0	0	0	**1**
1	0	0	0	1	**0**
1	0	0	1	0	**1**
1	0	0	1	1	**0**
1	0	1	0	0	**0**
1	0	1	0	1	**0**
1	0	1	1	0	**0**
1	0	1	1	1	**0**
1	1	0	0	0	**1**
1	1	0	0	1	**0**
1	1	0	1	0	**1**
1	1	0	1	1	**0**
1	1	1	0	0	**0**
1	1	1	0	1	**0**
1	1	1	1	0	**0**
1	1	1	1	1	**0**

- **TELasa**= (!Snai2 & !ESE2) | (Snai2 & !ESE2)

Snai2	ESE2	**TELasa**
0	0	**1**
0	1	**0**
1	0	**1**
1	1	**0**

- **Rb=** (!Cyclin & !p16 & p53) | (!Cyclin & p16 & !p53) | (!Cyclin & p16 & p53) | (Cyclin & !p16 & p53) | (Cyclin & p16 & !p53) | (Cyclin & p16 & p53)

Cyclin	p16	P53	**Rb**
0	0	0	**0**
0	0	1	**1**
0	1	0	**1**
0	1	1	**1**
1	0	0	**0**
1	0	1	**1**
1	1	0	**1**
1	1	1	**1**

- **E2F**= (!Rb & !p53 & !Snai2 & !Cyclin) | (!Rb & !p53 & !Snai2 & Cyclin)

Rb	p53	Snai2	Cyclin	**E2F**
0	0	0	0	**1**
0	0	0	1	**1**
0	0	1	0	**0**
0	0	1	1	**0**
0	1	0	0	**0**
0	1	0	1	**0**
0	1	1	0	**0**
0	1	1	1	**0**
1	0	0	0	**0**
1	0	0	1	**0**
1	0	1	0	**0**
1	0	1	1	**0**
1	1	0	0	**0**
1	1	0	1	**0**
1	1	1	0	**0**
1	1	1	1	**0**
